# Sex-Specific Sarcopenia Prevalence and Risk Factors in the Korean Population: A Cross-Sectional Epidemiological Study

**DOI:** 10.3390/medicina60060899

**Published:** 2024-05-29

**Authors:** Do-Youn Lee

**Affiliations:** College of General Education, Kookmin University, Seoul 02707, Republic of Korea; triptoyoun@kookmin.ac.kr; Tel.: +82-02-910-5540

**Keywords:** sarcopenia, prevalence, risk factors

## Abstract

*Background and Objective:* This study aimed to identify the incidence of sarcopenia and disease risk factors in Korean adults and to provide data for sarcopenia prevention. *Materials and Methods:* Based on the Korea National Health and Nutrition Survey, 2008–2011, we selected 14,185 adults over the age of 20 who participated in sarcopenia diagnostic tests and health surveys. We analyzed sarcopenia risk factors using complex sample multi-logistic regression analysis. *Results:* The prevalence of sarcopenia in Korea was 31.3%, with 20.2% in men and 40.4% in women. In men, there was a higher risk of sarcopenia in those of older age, without a spouse, with a low body mass index (BMI), who never engage in resistance exercise, or who do mid-level intensity resistance exercises. In women, sarcopenia risk was higher in those in their 20s compared to those in their 60s, and risk factors included a low BMI, high-density lipoprotein cholesterol and waist circumference measurements, alcohol consumption, aerobic exercise, and resistance exercise. *Conclusions:* Interventions and lifestyle improvements will help prevent the onset of sarcopenia in elderly men and young women with risk factors such as a low BMI.

## 1. Introduction

Sarcopenia involves the loss of muscle mass, muscle strength, and physical performance due to a lack of exercise, aging, and hormonal changes, although a clear mechanism has not yet been identified [1]. The loss of muscle strength caused by sarcopenia leads to mobility limitations and decreased physical activity levels [2,3], and decreased muscle mass is associated with cardiopulmonary vascular disease, osteoporosis, metabolic syndrome, physical damage, and high mortality [4,5,6,7]. Furthermore, there is a 1–2% decrease in skeletal muscle per year after the age of 35 and a 3% decrease per year after the age of 65 [8].

Sarcopenia shows differences in prevalence between men and women, but mainly after their 40s, as muscle mass begins to decrease and shows a rapid decrease with age. Several previous studies have reported that the prevalence of sarcopenia in the elderly (over 65 years of age) is about 10% and increases to up to 50% or more after 80 years of age [9,10]. Sarcopenia can cause falls, limit daily activities, and reduce quality of life [10,11,12].

Factors related to sarcopenia include nutritional intake, physical activity, alcohol consumption, and smoking status [13,14], and high-quality nutritional status and exercise are known to be key factors in preventing sarcopenia [15]. Recently, as interest in sarcopenia has increased in Korea, active research has been conducted and the prevalence of sarcopenia in elderly men has been found at 9.7% and 11.8% in elderly women [16].

An early diagnosis of sarcopenia allows for prompt and effective treatment [17]. Delayed treatment deteriorates a patient’s quality of life, increases health problems, and raises treatment costs [18]. Identifying disease risk factors helps to prevent sarcopenia, which reduces this cost burden, and it is important to shift the paradigm to prevention rather than treatment.

Most sarcopenia studies in Korea focus on individual factors, such as the association between sarcopenia and certain diseases, physical activity, and walking, and few studies have analyzed multiple risk factors [19,20,21,22]. Moreover, studies on risk factors for sarcopenia are mostly limited to elderly participants [23,24]. Sarcopenia risk factors may differ in men and women, but the analysis of factors related to sarcopenia for overall age is limited by sex [19,21,22,23,24]. 

This study supports the development of sarcopenia prevention programs by monitoring sarcopenia prevalence and risk factors in Korean adults using national statistical data. It examines the role of sex in sarcopenia prevalence and risk factors.

## 2. Materials and Methods

**Figure 1:**
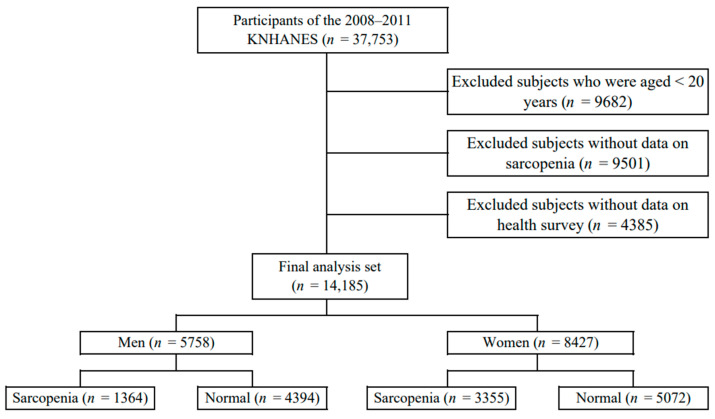
Selection of participants from the Korea National Health and Nutrition Examination Survey 2008–2011.

This study used data from 2008 to 2011 from the Korea National Health and Nutrition Examination Survey (KNHANES) conducted by the Korea Centers for Disease Control and Prevention. The subjects were selected as those who measured whole-body dual-energy X-ray absorptiometry (DXA) for the diagnosis of sarcopenia among adults over 20 years of age. Among them, subjects who participated in both the health survey and the physical measurement test were selected. Of the 37,753 subjects who participated in the survey, 9682 subjects under the age of 20, 9501 subjects without sarcopenia, and 4385 participants without the health survey and physical measurement test were excluded. Finally, 14,185 subjects were selected (Figure 1).

### 2.1. Demographic and Sociological Factors

As for demographic and sociological variables, items such as gender, age, education level, marital status, and personal income level were collected. Age was divided into 50s, 60s, and 70s or older. Education level was divided into low or high based on high school graduation. Marriage status was classified according to whether the participant lived with their current spouse. The average monthly individual income was divided using a quartile.

### 2.2. Health and Disease-Related Factors

Health and disease-related variables were collected: height, weight, body mass index (BMI), blood pressure, blood sugar, triglyceride, HDL-C, waist circumference, smoking and drinking status, and aerobic and resistance exercise. BMI was calculated by dividing body weight (kg) by the square of height (m^2^). It was divided into low weight, normal, overweight, and obesity. Blood pressure was measured using a mercury hypertension meter, and after 5 min of rest using a cuff suitable for the arm circumference, it was measured three times at 30 s intervals. Hypertension was classified as systolic blood pressure of 130 mmHg or more, diastolic blood pressure of 85 mmHg or more, or if the participant was currently taking antihypertensive drugs. Blood tests were collected while maintaining an empty stomach for more than 8 h and analyzed within 24 h using a Hitachi Automatic Analyzer 7600 (Hitachi, Tokyo, Japan). Hyperglycemia was defined as the case of fasting blood glucose ≥ 100 mg/dL or the participant taking diabetic drugs. Hypertriglyceridemia was classified as triglyceride of 150 mg/dL or more. Low HDL-C was classified as less than 40 mg/dL in men and less than 50 mg/dL in women, and abdominal obesity was classified as more than 90 cm in men and 85 cm in women based on waist circumference (WC) [5].

Smoking status was classified as ‘daily smoking’ and ‘occasionally smoking’ as current smoking, ‘smoking in the past, but not now’ as past smoking, and ‘never smoked’ as non-smoking. Drinking status was classified as current drinking in response to ‘more than once a month’ and ‘less than once a month’ and non-drinking in response to ‘not drinking at all in the last year’.

Aerobic exercise was determined as the walking time, as follows. Number of days the subject walked ≥10 min at a time for the last 1 week was expressed. Walking was measured by total walking time in a week (TWT), calculated as follows: TWT = walking days (days/week) × walking minutes (minutes/day). Frequency of resistance exercise was assessed according to participants’ answers to the question “How many times do you do resistance exercise (push-ups, sit-ups, lifting dumbbells or barbells) a week?” If there was no resistance exercise at all, it was classified into a medium intensity group for 1 to 3 days of resistance exercise, and a high intensity group for more than 4 days. 

### 2.3. Sarcopenia Measurement

Sarcopenia-related body composition was measured by licensed technicians using DXA (Discovery QDR 4500 W, Hologic Inc., Belford, MA, USA). Participants fasted prior to the assessment and were in the supine position during the assessment. All non-fat and non-bone tissue was assumed to be skeletal muscle. 

Appendicular skeletal muscle mass (ASM) was calculated as the sum of skeletal muscle mass in both arms and legs, as measured by DXA. The subjects’ skeletal muscle mass index (SMI) was calculated as their ASM (kg) divided by their height in meters squared (m^2^). Sarcopenia was defined as SMI values <7.0 kg/m^2^ for men and <5.4 kg/m^2^ for women, as recommended by the Asian Working Group for Sarcopenia (AWGS) [25].

### 2.4. Data Analysis

The data were analyzed using SPSS 27.0 window version (IBM, Armonk, NY, USA). The responses were weighted by reference to the multistage, complex, probability sampling design. Data are expressed as absolute numbers and estimated percentages (with standard errors (SE)). The specific analysis method is as follows. First, the difference in characteristics between sarcopenia and the normal group was analyzed by t-test and complex sample cross-analysis (χ^2^-test). Second, complex sample multiple logistic regression analysis was used to analyze the risk factors affecting sarcopenia, and statistics were expressed as odds ratio (OR) and 95% confidence interval (CI).

## 3. Results

### 3.1. Prevalence of Sarcopenia and Demographic Sociological Characteristics According to Sex

**Figure 2:**
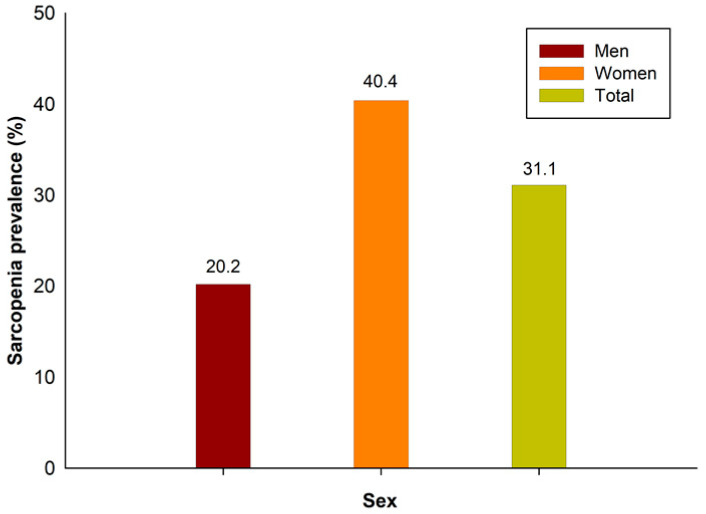
Sarcopenia prevalence in Korea.

The prevalence of sarcopenia in this study was 31.1%: 20.2% in men and 40.4% in women (Figure 2). The demographic and sociological characteristics of the participants are presented in Table 1 and Table 2. In men, there were statistically significant differences between those with and without sarcopenia in all variables, except for marital status, individual income, smoking status, and aerobic exercise. In women, there were statistically significant differences in all variables except for marital status, height, smoking status, and alcohol status.

### 3.2. Multiple Logistic Regression Analysis for Sarcopenia Risk Factor

Table 3 and Table 4 show the sarcopenia risk factors according to sex. In Table 3, per simple logistic regression analysis, the factors affecting sarcopenia in men were age, individual income, BMI, blood pressure, blood glucose level, TG, HDL-C, WC, and aerobic and resistance exercises. In Table 3, the Nagelkerke R2 = 0.335 in the final multiple logistic regression analysis model. After adjusting for covariates in the multiple logistic regression analysis, the sarcopenia risk factors for men were age, individual income, BMI, HDL-C, WC, alcohol status, and levels of aerobic and resistance exercise. The ORs were 3.201 (95% CI 1.824–5.618) and 6.393 (95% CI 3.691–11.073), respectively, for individuals in their 60s and 70s compared to those in their 20s. For BMI and WC, which are obesity indicators, sarcopenia prevalence was higher in underweight individuals and lower in overweight or obese individuals. For resistance exercise, those who performed resistance exercise and those in the mid-intensity group had ORs of 1.808 (95% CI 1.404–2.329) and 1.664 (95% CI 1.178–2.251), respectively, compared to the high-intensity group. The results indicate that men in their 60s and 70s who are underweight and who do not perform resistance exercises have a higher prevalence of sarcopenia.

A simple logistic regression analysis (Table 4) shows that the factors affecting sarcopenia in women were age, educational level, BMI, blood pressure, blood glucose, TG, HDL-C, WC, and aerobic and resistance exercise levels. After adjusting for covariates, the sarcopenia risk factors in women were age, individual income, BMI, HDL-C, WC, alcohol status, and resistance exercise. Those in their 60s had an OR of 0.661 (95% CI 0.469–0.931) compared to women in their 20s. Regarding individual income, those in Q1 had an OR of 0.819 (95% CI 0.686–0.979) compared to those in the Q4 quartile. Sarcopenia prevalence was higher in those with high BMI and WC compared to underweight individuals. Those with a low HDL-C had an OR of 0.786 (95% CI 0.690–0.895) compared to those with a normal HDL-C level. Those who never performed resistance exercise had an OR of 1.644 (95% CI 1.252–2.159) compared to the high-intensity group. Sarcopenia prevalence was high in women in their 20s and those who were underweight, with high HDL-C levels, and who never performed resistance exercise.

## 4. Discussion

The purpose of this study was to investigate sarcopenia prevalence and risk factors by sex, as well as to promote sex-specific care and prevention techniques. In this study, the prevalence of sarcopenia among adults in Korea was 31.1%: 20.4% in men and 40.4% in women.

The results of this study indicate that men in their 60s and 70s who are underweight and who do not perform resistance exercises have a higher prevalence of sarcopenia. In men, the risk of sarcopenia was 3.201 times higher for those in their 60s and 6.393 times higher for those in their 70s compared to individuals in their 20s. This was similar to the results of a previous study, in which limb muscle mass significantly decreased in men with age [26]. 

In women, a univariate logistic analysis showed that sarcopenia prevalence was significantly higher in individuals in their 20s compared to any other age group. A multivariate analysis showed that the prevalence of sarcopenia for women in their 20s was significantly higher than for women in their 60s. This is contrary to several previous studies showing that sarcopenia prevalence is high in elderly populations [23,27,28]. 

There may be several reasons why sarcopenia is more common in women in their 20s compared to women in their 60s. The blatant stigma of obesity [29] and the influence of body dissatisfaction [30] through mass media has spread the culture of lookism [31]. Korean women often consume a strict diet regardless of their weight, especially those in their 20s [32,33]. The obesity rate of Korean men is 22.6% in their 20s and 31.1% in their 60s, while that of women is about 3.4 times different, 8.6% in their 20s and 29.0% in their 60s [34]. Due to this effect, it is thought that women have a greater risk of sarcopenia in their 20s.

Regardless of sex, the sarcopenia prevalence was significantly higher in underweight individuals and those who never engaged in resistance exercise. Several previous studies have shown that SMI and BMI are strongly correlated, and BMI tends to decrease in patients with sarcopenia [35,36,37]. Sarcopenia prevalence in women with obesity was lower than in non-obese women [38]. A low BMI can be said to be one of the risk factors for sarcopenia.

Several studies have shown that physical activity levels are highly correlated with sarcopenia, particularly resistance exercise [39,40,41]. A previous study found that the sarcopenia rate was significantly higher in subjects with long-term sedentary behavior, and sedentary behaviors lasting more than seven days result in a loss of 30% of the muscle mass [14,39,40]. The results of this study also show that men are 1.664 times more likely to have sarcopenia if they are in the mid-intensity exercise group and 1.808 times more likely if they never engage in resistance exercise compared to those in the high-intensity group, consistent with previous studies. In women, there was no significant difference in sarcopenia risk between those in the high- and mid-intensity groups, but the prevalence of sarcopenia was 1.644 times higher in those who never performed resistance exercise compared to those in the high-intensity group.

In the results of this study, HDL-C levels for men and women were higher in the sarcopenia group (at 47.83 ± 0.42 mg/dL and 52.22 ± 0.25 mg/dL, respectively) compared to the normal group, but this difference was significant only in women in the multiple regression analysis that considered various covariates. This is similar to the results of previous studies, which found a higher incidence of sarcopenia with higher HDL-C levels [42,43,44]. However, since these preceding studies did not separate male and female groups, it is confirmed that there is some difference from the results in this study. Therefore, the fact that HDL-C showed a significant difference only in women is considered to require further research in the future.

This study has several limitations. First, it is a cross-sectional study that identified sarcopenia prevalence and risk factors, so there are limits in establishing a causal relationship. Future studies are necessary to confirm a causal relationship. Second, in the subjects of the KNHANES survey, the non-participation of a small number of severe sarcopenia patients may affect the outcome analysis. However, since these data were obtained for the national population, it is thought that the disturbing factors for a small number of people did not significantly affect the results. Third, the study data were from 12 years ago, and may not reflect the current population. However, this study may serve as a meaningful foundation for subsequent studies. Despite these limitations, this study has research significance as primary evidence for health promotion projects for subjects with sarcopenia.

## 5. Conclusions

This study was conducted to provide data for sarcopenia prevention and management by identifying the prevalence and risk factors according to sex in Korean adults. In this study, the overall prevalence of sarcopenia was 31.3%: 20.2% in men and 40.4% in women. The incidence of sarcopenia was high in older men, those who did not live with a spouse, were underweight, never performed resistance exercises, or who performed mid-intensity resistance exercises. On the other hand, in women, the risk of developing sarcopenia in their 20s was high, and low BMI, HDL-C, WC, alcohol consumption, and aerobic and resistance exercise were found to be risk factors. Therefore, sarcopenia risk factors must consider these risk factors according to sex when developing health-related education programs.

## Figures and Tables

**Table 1:** Characteristics in subjects according to sarcopenia in men.

Factors	Categories	Sarcopenia (*n* = 1364)		Normal (*n* = 4394)		χ^2^	*p* for Trend
		U/F	W/F	U/F	W/F		
		N or M	%	N or M	%		
Age	Total	53.77 ± 0.61		47.05 ± 0.32			<0.0001
	20–29	31	4.8	161	6.9	332.097	<0.0001
	30–39	155	16.8	892	24.0		
	40–49	178	20.7	1068	29.0		
	50–59	218	19.1	973	23.0		
	60–69	320	18.3	844	11.6		
	≥70	462	20.3	156	5.5		
Education	Low	834	54.4	1978	41.2	65.955	<0.001
	High	530	45.6	2416	58.8		
Marital status	With	1233	88.4	4090	90.8	5.795	0.086
	Without	131	11.6	304	9.2		
Individual income	Q1 (Lowest)	353	27.7	1011	24.1	9.994	0.056
	Q2	365	27.0	1163	27.0		
	Q3	351	25.0	1106	24.9		
	Q4 (Highest)	295	20.3	1114	24.0		
Height (cm)		168.14 ± 0.25		170.23 ± 0.13			<0.001
Weight (kg)		59.38 ± 0.31		72.33 ± 0.2			<0.001
BMI (kg/m^2^)	Index	20.98 ± 0.09		24.93 ± 0.06			<0.001
	Low	165	12.4	14	0.4	1036.097	<0.0001
	Normal	1144	82.9	2374	53.5		
	Overweight	55	4.6	1855	42.1		
	Obesity	0	0	151	4.0		
Blood pressure (mmHg)	Systolic	121.37 ± 0.63		121.81 ± 0.34			<0.001
	Diastolic	77.84 ± 0.38		81.08 ± 0.25			<0.001
	Hypertension	562	37.3	1951	42.1	8.807	0.009
Fasting glucose (mg/dL)		100.49 ± 1.06		100.91 ± 0.49			0.726
	Diabetes	496	34.5	1702	35.9	7.905	0.015
TG		145.55 ± 4.59		170.18 ± 2.72			<0.001
	High	394	30.7	1869	42.8	56.244	<0.001
HDL-C		47.83 ± 0.42		45.14 ± 0.23			<0.001
	Low	405	29.2	1632	35.1	14.759	<0.001
WC (cm)		78.17 ± 0.3		86.5 ± 0.17			<0.001
	Abdominal obesity	117	7.6	1474	32.1	282.883	<0.0001
SMI		6.51 ± 0.02		8.05 ± 0.02			<0.0001
Smoking status	Current	847	64.0	2859	66.3	2.404	0.405
	Past	294	20.0	859	18.4		
	Non	223	16.1	676	15.32		
Alcohol status	Yes	1030	80.8	3759	87.9	39.729	<0.001
	No	334	19.2	635	12.1		
Aerobic exercise	TWT	402.03 ± 21.18		384.09 ± 10.70			0.433
Resistance exercise	Never	1064	75.4	2962	67.5	31.600	<0.001
	Mid	158	15.1	738	17.5		
	High	142	9.5	694	15.0		

Data were presented as means ± SE (%). BMI, body mass index; BP, blood pressure; HDL-C, high-density lipoprotein cholesterol; WC, waist circumference; SMI, skeletal muscle mass index.

**Table 2:** Characteristics in subjects according to sarcopenia in women.

Factors	Categories	Sarcopenia (*n* = 3355)		Normal (*n* = 5072)		χ^2^	*p* for Trend
		U/F	W/F	U/F	W/F		
		N or M	%	N or M	%		
Age	Total	47.58 ± 0.37		50.17 ± 0.28			<0.001
	20–29	182	9.1	168	5.2	144.665	<0.0001
	30–39	851	26.2	895	19.8		
	40–49	724	25.2	1087	26.3		
	50–59	617	18.4	1140	22.5		
	60–69	483	9.7	1046	15.2		
	≥70	498	11.5	736	11.0		
Education	Low	1819	50.5	3259	60.5	81.447	<0.001
	High	1536	49.5	1813	39.5		
Marital status	With	2618	78.5	3922	78.7	0.072	0.824
	Without	737	21.5	1150	21.3		
Individual income	Q1 (Lowest)	753	24.6	1361	27.8	22.283	0.003
	Q2	824	25.0	1334	26.2		
	Q3	881	25.3	1253	24.8		
	Q4 (Highest)	897	25.0	1124	21.2		
Height (cm)		156.58 ± 0.15		156.48 ± 0.12			0.653
Weight (kg)		52.25 ± 0.14		61.4 ± 0.18			<0.001
BMI (kg/m^2^)	Index	21.33 ± 0.06		25.07 ± 0.07			<0.001
	Low	332	10.5	21	0.4	1814.13	<0.0001
	Normal	2793	83.2	2662	53.3		
	Overweight	224	6.2	2033	38.9		
	Obesity	6	0.2	356	7.4		
Blood pressure (mmHg)	Systolic	114.28 ± 0.42		119.27 ± 0.36			<0.001
	Diastolic	73.06 ± 0.24		75.93 ± 0.21			<0.001
	Hypertension	876	22.5	1785	31.5	81.053	<0.001
Fasting glucose (mg/dL)		93.61 ± 0.39		97.97 ± 0.35			<0.001
	Diabetes	661	18.4	1536	28.3	108.605	<0.0001
TG		105.18 ± 1.55		124.99 ± 1.5			<0.001
	High	613	17.0	1399	26.0	93.151	<0.001
HDL-C		52.22 ± 0.25		49.11 ± 0.21			<0.001
	Low	1595	45.0	3018	57.4	124.893	<0.0001
WC (cm)		73.88 ± 0.2		82.68 ± 0.2			<0.001
	Abdominal obesity	336	9.0	2070	38.5	903.405	<0.0001
SMI		5.26 ± 0.01		6.38 ± 0.02			<0.0001
Smoking status	Current	293	9.6	390	8.7	3.006	0.422
	Past	91	3.3	103	2.9		
	Non	2971	87.1	4579	88.3		
Alcohol status	Yes	1981	62.8	3066	64.3	1.980	0.236
	No	1374	37.2	2006	35.7		
Aerobic exercise	TWT	274.38 ± 10.48		314.66 ± 8.58			0.002
Resistance exercise	Never	2960	88.3	4393	85.3	27.053	<0.001
	Mid	255	7.8	386	8.3		
	High	140	3.9	293	6.4		

Data were presented as means ± SE (%). BMI, body mass index; BP, blood pressure; HDL-C, high-density lipoprotein cholesterol; WC, waist circumference; SMI, skeletal muscle mass index.

**Table 3:** Multiple logistic regression analysis for sarcopenia risk factor in men.

Factors	Categories	Crude		Adjusted	
		OR (95% CI)	*p*-Value	OR (95% CI)	*p*-Value
Age	Total				
	20–29	1		1	
	30–39	1.009 (0.632–1.610)	0.971	1.254 (0.728–2.160)	0.414
	40–49	1.029 (0.641–1.651)	0.905	1.482 (0.860–2.555)	0.156
	50–59	1.193 (0.740–1.926)	0.469	1.662 (0.955–2.891)	0.072
	60–69	2.276 (1.419–3.652)	<0.001	3.201 (1.824–5.618)	<0.001
	≥70	5.357 (3.331–8.614)	<0.001	6.393 (3.691–11.073)	<0.001
Education	Low	1.704 (1.452–2.000)	<0.001	1.061 (0.864–1.304)	0.57
	High	1		1	
Individual income	Q1 (Lowest)	1.352 (1.082–1.689)	0.008	1.222 (0.874–1.441)	0.367
	Q2	1.179 (0.955–1.456)	0.126	1.165 (0.907–1.498)	0.232
	Q3	1.182 (0.953–1.466)	0.127	1.071 (0.830–1.381)	0.598
	Q4 (Highest)	1		1	
BMI (kg/m^2^)	Low	20.241 (10.280–39.851)	<0.001	20.675 (10.542–40.548)	<0.001
	Normal	1		1	
	Overweight	0.071 (0.048–0.105)	<0.001	0.093 (0.063–0.137)	<0.001
	Obesity	0.0001 (0.0002–0.0003)	<0.0001	0.001 (0.0004–0.0008)	<0.0001
Blood pressure (mmHg)	Normal	1		1	
	Hypertension	0.818 (0.704–0.952)	0.009	0.929 (0.779–1.107)	0.409
Fasting glucose (mg/dL)	Normal	1		1	
	Diabetes	0.821 (0.701–0.962)	0.015	0.970 (0.811–1.160)	0.738
TG	Normal	1		1	
	High	0.593 (0.504–0.697)	<0.001	1.039 (0.850–1.271)	0.709
HDL-C	Normal	1		1	
	Low	0.760 (0.651–0.888)	<0.001	0.985(0.803–1.209)	0.888
WC (cm)	Normal	1		1	
	Abdominal obesity	0.174 (0.135–0.224)	<0.001	0.616 (0.459–0.827)	0.001
Alcohol status	Yes	0.581 (0.485–0.695)	<0.001	0.851 (0.681–1.063)	0.313
	No	1		1	
Aerobic exercise	TWT	1.001 (0.999–1.002)	0.418	1.000 (0.998–1.001)	0.55
Resistance exercise	Never	1.757 (1.391–2.218)	<0.001	1.808 (1.404–2.329)	<0.001
	Mid	1.353 (0.998–1.833)	0.051	1.664 (1.178–2.351)	0.004
	High	1		1	

Data were presented as means ± SE (%). BMI, body mass index; BP, blood pressure; HDL-C, high-density lipoprotein cholesterol; WC, waist circumference.

**Table 4:** Multiple logistic regression analysis for sarcopenia risk factor in women.

Factors	Categories	Crude		Adjusted	
		OR (95% CI)	*p*-Value	OR (95% CI)	*p*-Value
Age	Total				
	20–29	1		1	
	30–39	0.600 (0.462–0.781)	0.031	0.867 (0.644–1.167)	0.346
	40–49	0.371 (0.286–0.482)	<0.001	0.766 (0.562–1.045)	0.092
	50–59	0.472 (0.363–0.613)	<0.001	0.736 (0.528–1.026)	0.07
	60–69	0.554 (0.432–0.710)	<0.001	0.661 (0.469–0.931)	0.018
	≥70	0.764 (0.599–0.975)	<0.001	0.941 (0.663–1.337)	0.736
Education	Low	0.668 (0.598–0.745)	<0.001	1.060 (0.907–1.238)	
	High	1		1	
Individual income	Q1 (Lowest)	0.749 (0.637–0.880)	<0.001	0.819 (0.686–0.979)	0.028
	Q2	0.810 (0.694–0.946)	0.008	0.844 (0.708–1.005)	0.056
	Q3	0.865 (0.739–1.013)	0.072	0.861 (0.729–1.017)	0.079
	Q4 (Highest)	1		1	
BMI (kg/m^2^)	Low	15.631 (8.405–29.070)	<0.001	13.309 (7.088–24.990)	<0.001
	Normal	1		1	
	Overweight	0.102 (0.084–0.123)	<0.001	0.133 (0.107–0.165)	<0.001
	Obesity	0.018 (0.007–0.045)	<0.001	0.028 (0.011–0.072)	<0.001
Blood pressure (mmHg)	Normal	1		1	
	Hypertension	0.633 (0.563–0.711)	<0.001	1.008 (0.872–1.166)	0.91
Fasting glucose (mg/dL)	Normal	1		1	
	Diabetes	0.570 (0.504–0.646)	<0.001	1.035 (0.882–1.214)	0.519
TG	Normal	1		1	
	High	0.586 (0.514–0.668)	<0.001	1.055 (0.896–1.244)	0.537
HDL-C	Normal	1		1	
	Low	0.607 (0.549–0.673)	<0.001	0.786 (0.690–0.895)	<0.001
WC (cm)	Normal	1		1	
	Abdominal obesity	0.158 (0.136–0.184)	<0.001	0.617 (0.503–0.757)	<0.001
Alcohol status	Yes	0.937 (0.842–1.043)	0.236	0.858 (0.751–0.980)	0.02
	No	1		1	
Aerobic exercise	TWT	0.998 (0.997–0.999)	0.005	1.001 (0.999–1.002)	0.056
Resistance exercise	Never	1.718 (1.350–2.186)	<0.001	1.644 (1.252–2.159)	<0.001
	Mid	1.567 (1.150–2.137)	0.005	1.351 (0.965–1.892)	0.079
	High	1		1	

Data were presented as means ± SE (%). BMI, body mass index; BP, blood pressure; HDL-C, high-density lipoprotein cholesterol; WC, waist circumference.

## Data Availability

All data were anonymized and can be downloaded from the website (https://knhanes.kdca.go.kr/knhanes, accessed on 10 March 2024).
